# Circulating MicroRNAs as Easy-to-Measure Aging Biomarkers in Older Breast Cancer Patients: Correlation with Chronological Age but Not with Fitness/Frailty Status

**DOI:** 10.1371/journal.pone.0110644

**Published:** 2014-10-21

**Authors:** Sigrid Hatse, Barbara Brouwers, Bruna Dalmasso, Annouschka Laenen, Cindy Kenis, Patrick Schöffski, Hans Wildiers

**Affiliations:** 1 Laboratory of Experimental Oncology (LEO), Department of Oncology, KU Leuven, and Department of General Medical Oncology, University Hospitals Leuven, Leuven Cancer Institute, Leuven, Belgium; 2 Department of Internal Medicine, Istituto di Ricerca a Carattere Clinico e Scientifico (IRCCS), Azienda Ospedaliera Universitaria (AOU) San Martino Istituto Nazionale Tumori (IST), Genoa, Italy; 3 Interuniversity Centre for Biostatistics and Statistical Bioinformatics, Leuven, Belgium; 4 Department of Geriatric Medicine, University Hospitals Leuven, Leuven, Belgium; University of Valencia, Spain

## Abstract

Circulating microRNAs (miRNAs) hold great promise as easily accessible biomarkers for diverse (patho)physiological processes, including aging. We have compared miRNA expression profiles in cell-free blood from older versus young breast cancer patients, in order to identify “aging miRNAs” that can be used in the future to monitor the impact of chemotherapy on the patient’s biological age. First, we assessed 175 miRNAs that may possibly be present in serum/plasma in an exploratory screening in 10 young and 10 older patients. The top-15 ranking miRNAs showing differential expression between young and older subjects were further investigated in an independent cohort consisting of another 10 young and 20 older subjects. Plasma levels of miR-20a-3p, miR-30b-5p, miR106b, miR191 and miR-301a were confirmed to show significant age-related decreases (all p≤0.004). The remaining miRNAs included in the validation study (miR-21, miR-210, miR-320b, miR-378, miR-423-5p, let-7d, miR-140-5p, miR-200c, miR-374a, miR376a) all showed similar trends as observed in the exploratory screening but these differences did not reach statistical significance. Interestingly, the age-associated miRNAs did not show differential expression between fit/healthy and non-fit/frail subjects within the older breast cancer cohort of the validation study and thus merit further investigation as true aging markers that not merely reflect frailty.

## Introduction

Given the increasing proportion of older people in the general population, oncologists nowadays encounter the great challenge of proper cancer management in older patients. A major problem is the heterogeneous health condition among older cancer patients, making it difficult to rely on standard treatment guidelines established for distinct cancer types and, hence, demanding a more individualized approach. Secondly, long-term impact of chemotherapy on the patient’s global health condition may be more pronounced and, at the same time, less predictable in older individuals.

Body aging is a complex phenomenon involving several, partly overlapping, molecular mechanisms. Among others, these include the accumulation of oxidative stress [1] and DNA damage [2], the shortening of telomeres [3], [4]and neuroendocrine and immunologic changes [5], [6].

It is generally assumed that chemotherapy may accelerate the aging process through interference at the level of one or several of these aging driving forces [7]. For example, free radical intermediates are generated during the metabolism anthracyclines, alkylating agents directly cause DNA damage, while topoisomerase inhibitors, such as epirubicin, inhibit DNA repair enzymes. Also, chemotherapy may accelerate leukocyte telomere attrition, due to telomerase inhibition [8] and/or repeated cycles of intense hematological repopulation [9]–[11]. Moreover, chemoradiotherapy may have more severe effects on the replicative capacity of blood cells in older as compared to younger patients [12]. Finally, neuroendocrine and immune functions can also be affected by chemotherapeutic agents and by corticosteroids that are often incorporated in chemotherapeutic regimens [7].

At the cellular level, aging is intimately linked with tumorigenesis through the mechanism of cellular senescence [13], a cancer-protective stress response that causes irreversible growth arrest in order to prevent further proliferation of damaged, potentially harmful cells. While being a potent anti-tumor mechanism early in life, cellular senescence is at the same time responsible for tissue aging at older age, through accumulation of non-proliferative cells [13], finally resulting in tissue dysfunction and age-related diseases. Standard chemotherapy and radiotherapy might function in part by inducing senescence within the tumor mass [14], [15], but might concurrently cause increased senescence induction in healthy, proliferating tissues as well.

Taken together, chemotherapeutic treatment may potentially lead to accelerated aging and/or premature onset of frailty in older cancer patients [7]. Therefore, the patient’s biological aging profile is of particular relevance in geriatric oncology and may aid in oncological decision making and individual treatment optimization. Several aging biomarkers have already been described, most particularly mean leukocyte telomere length and p16^INK4a^ mRNA expression in T lymphocytes [16]–[18]. However, none of these so far made its way into routine clinical practice and the search for robust and easy-to-measure aging biomarkers is still ongoing.

MicroRNA’s (miRNAs) are short (20–24 nt), non-coding and highly stable RNA’s that are involved in post-transcriptional regulation of gene expression. They are known as fine-tuning mediators of a wide variety of normal physiological pathways, developmental processes and pathological conditions; it is thus plausible that they also play a role in cellular senescenceand tissue/body aging [19], [20]. Several miRNAs involved in DNA damage response, cellular senescence and cell death (such as let-7, miR-34 and miR-43), were indeed identified in the widely used *C. elegans* aging model and turned out to be highly conserved among species [21]. In aging mice, miR-34a was shown to be upregulated, concomitantly with a decreased mRNA expression of its primary target gene SIRT1, in brain tissue but also in peripheral blood mononuclear cells and plasma [22]. Therefore, it was suggested that circulatory miR-34a may hold great promise as an accessible biomarker for brain aging.

Circulating miRNAs actually are attractive candidate biomarkers for clinical use, because of their easy accessibility and outstanding stability in serum/plasma [23]. Here, we describe the potential use of microRNA signatures expressed in serum/plasma for the assessment of biological age in breast cancer patients. We compared a panel of 175 different microRNAs, known to be among the most relevant in serum/plasma, between older and young breast cancer patients and validated the findings of this initial exploratory screening in an independent breast cancer cohort. At least 5 circulating microRNAs emerged from this study that are worth to be further explored as potential aging biomarkers in larger cohorts of young *versus* old fit *versus* old frail individuals, both within and beyond a cancer background.

## Results

**Figure 1:**
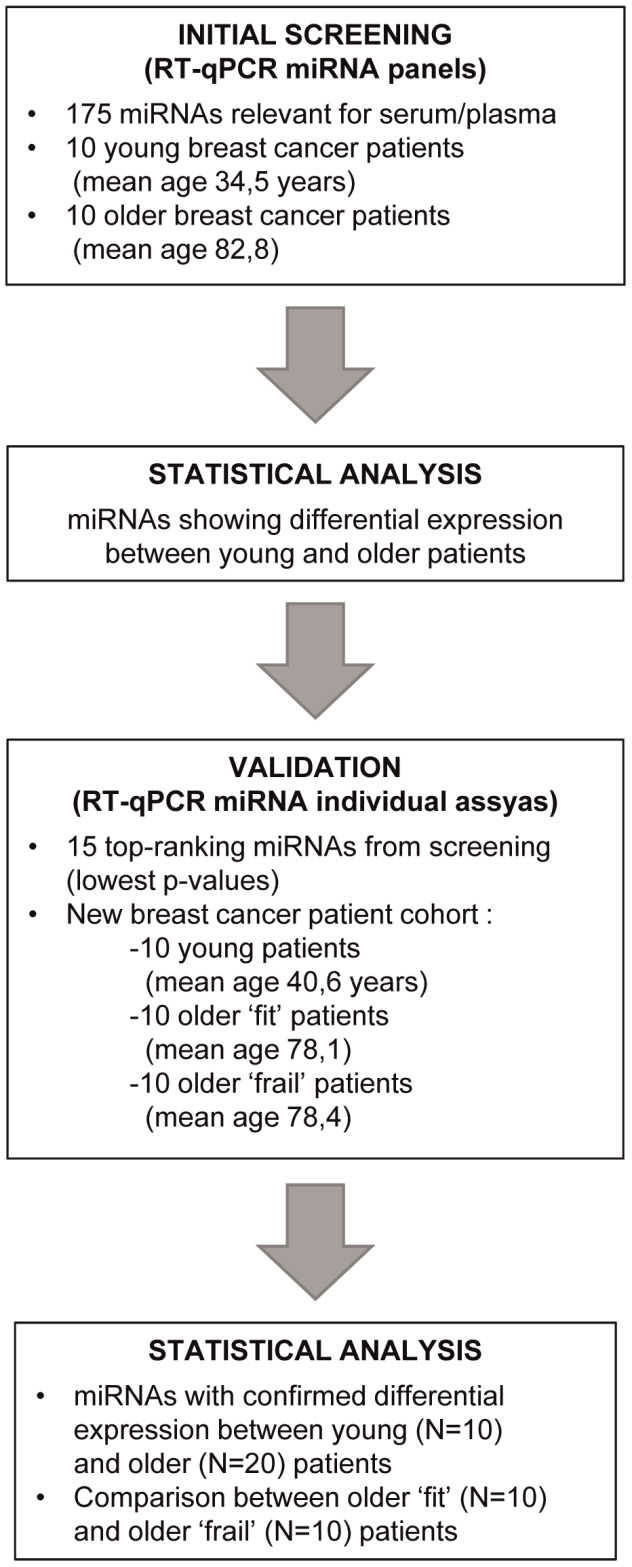
Experimental study design.

The experimental design of our study is shown in Fig. 1. In the first stage, the expression profiles of 175 miRNAs were analyzed in serum samples of 10 young (mean age 34.5 years) and 10 older (mean age 82.8 years) breast cancer patients. Distinct breast cancer subtypes were equally represented in both groups (see Table 1). From this initial screening experiment, the 15 top-ranking miRNAs (showing the highest significance for differential expression between both groups) were selected, together with 4 candidate reference miRNAs (showing stable expression among all samples). In the second stage, the selected potential aging miRNAs and candidate reference miRNAs were re-evaluated by individual RT-qPCR in plasma samples from a new, independent study cohort consisting of 10 young (mean age 41 years) and 20 older (mean age 78 years) breast cancer patients, all diagnosed with lymph node negative, luminal A (grade 1–2, ER-positive, PR-positive, HER2-negative) tumors (see Table 2). Geriatric assessment in the older cohort was performed as described by Kenis *et al.* [24].

**Table 1:** Patient and tumor characteristics of the pilot study cohort.

	Young group	Older group
	N = 10	N = 10
**Age at diagnosis**		
Mean (years)	34,5	82,8
Range (years)	28–38	80–89
**Breast cancer subtype**		
Luminal A-like phenotype	7	7
Luminal B-like phenotype	1	1
Triple negative-like phenotype	2	2
**Tumor grade**		
I	2	2
II	7	5
III	1	3
**Histological subtype**		
Invasive ductal carcinoma	9	7
Invasive lobular carcinoma	1	1
Other	0	2
**Estrogen receptor status**		
Positive	8	8
Negative	2	2
**Progesteron receptor status**		
Positive	7	8
Negative	3	2
**HER-2 receptor status**		
Positive	0	0
Negative	10	10
**Pathological staging (pT)**		
pT1a	1	0
pT1b	0	1
pT1c	4	1
pT2	4	8
x	1	0
**Nodal status**		
pN0	10	10

**Table 2:** Patient and tumor characteristics of the validation study cohort.

	Young group	Older ‘fit’ group	Older ‘non-fit’ group
	(N = 10)	(N = 10)	(N = 10)
**Age at diagnosis**			
Mean (years)	41	78	78
Range (years)	37–43	71–83	73–91
**Frailty status (Balducci)**			
Frail	N/A	0	9
Vulnerable	N/A	4	1
Fit	N/A	6	0
**Frailty status (LOFS** a **)**			
0–2 (severely frail)	N/A b	0	1
3–4 (frail)	N/A	0	1
5–6 (vulnerable)	N/A	0	8
7–8 (slightly vulnerable)	N/A	0	0
9–10 (fit)	N/A	10	0
**Breast cancer subtype**			
Luminal A-like	10	10	10^c^
**Tumor grade**			
I	4	2	2
II	6	8	8
III	0	0	1^c^
**Histological subtype**			
Invasive ductal carcinoma	8	6	5
Invasive lobular carcinoma	2	3	2
Other	0	1	3
**Estrogen receptor status**			
Positive	10	10	10
Negative	0	0	0
**Progesteron receptor status**			
Positive	10	10	10
Negative	0	0	0
**HER-2 receptor status**			
Positive	0	0	0
Negative	10	10	10
**Pathological staging (pT)**			
pT1a	0	0	0
pT1b	4	1	2
pT1c	2	3	1
pT2	4	5	7
pT3	0	1	0
**Nodal staging (pN)**			
pN0	10	10	10

a Leuven Oncology Frailty Score : refer to File S1 for more details.

b N/A : not applicable.

c One patient had a mixed luminal A (grade II)/luminal B (grade III) tumor.

### Exploratory screening for differentially expressed miRNAs in older versus young breast cancer patients

Profiles of circulating miRNAs were analyzed in the 10 young and 10 older breast cancer patients of the exploratory cohort using the Exiqon miRNA serum/plasma focus panels, including 175 different miRNAs (refer to File S2 for a complete list of included miRNAs). Plate-to-plate variations in qPCR efficiency were small: the standard deviation of Cp values obtained for the individual interplate calibrator wells was 0.33 cycles, and no outliers (deviation of 4 times the standard deviation or more) were detected. Comparable RNA extraction and reverse transcription efficiency across all samples of the cohort was ascertained by the UniSp6 spike-in control : standard deviation of the UniSp6 PCR Cp values (after interplate calibration) was 0.55 cycles (mean Cp was 25,99) and no outliers were detected, indicating that no inhibitors of reverse transcription and/or PCR reactions were present. The average signal of all miRNAs was comparable in both age categories : the global mean Cp of the young patient group was 33.80±2.01, compared to 33.90±1.67 for the old group. Thus, there were no significant age-related changes in total miRNA content of the serum samples.

**Figure 2:**
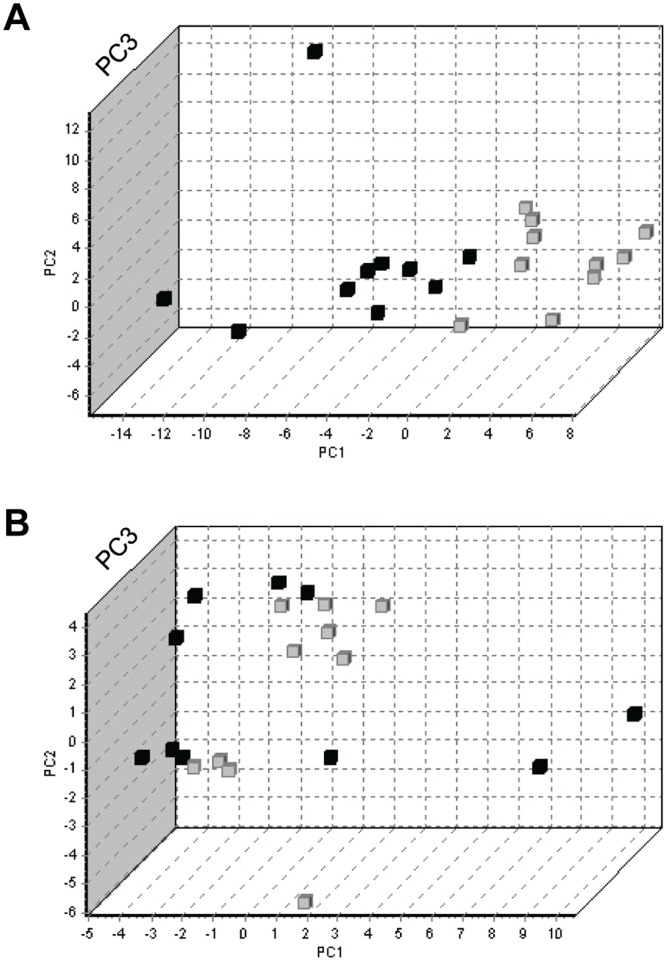
Principal component analysis (PCA) of the panel screening results. (**A**) PCA analysis of the top-50 ranking (lowest P-values in t-test) miRNAs of the panel screening; (**B**) PCA analysis of the bottom-50 ranking miRNAs (highest P-values in t-test). Black symbols represent young patients; grey symbols represent older, frail patients. PC1, PC2 and PC3 on X, Y and Z-axis represent the 3 principal components generated by this statistical algorithm.

**Figure 3:**
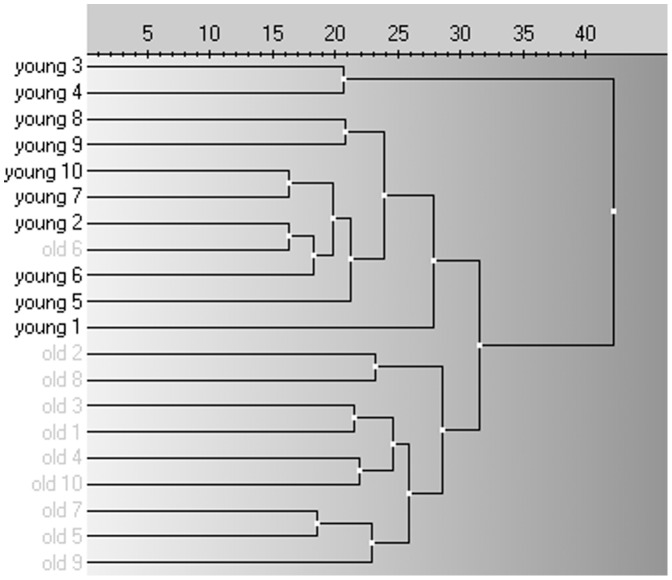
Dendrogram showing hierarchical clustering of patients according to the exploratory miRNA screening panel results. Young patients are shown in black, older patients are shown in grey. The clustering was performed on all 20 patients of the exploratory cohort and on the entire miRNA screening panel, applying complete linkage as the clustering method and Euclidean distance as the distance measure.

**Figure 4:**
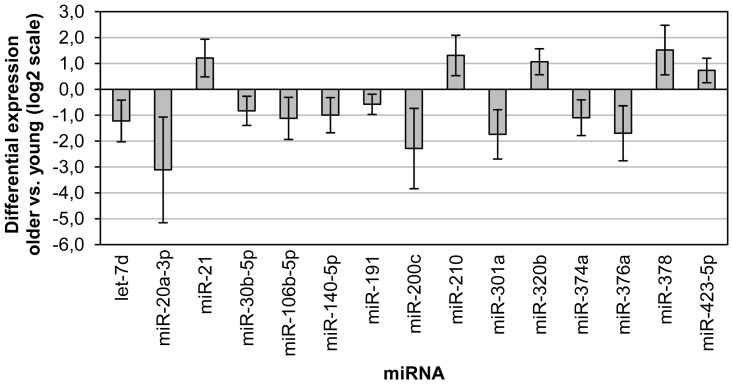
Differential expression of the top-15 ranking miRNAs from the panel screening. The selected miRNAs are displayed in numerical order. Bars represent the difference in expression in older compared to young patients on a 2-logarithmic scale, with indication of the 95% confidence interval (based on t-test).

After global mean normalisation (i.e. normalisation to the average signal of all miRNAs included in the panel) [25], statistical analysis of the results revealed 37 miRNAs with significantly different (p<0.05) expression in serum samples from the older compared to the young patient group. The first 50 miRNAs of the t-test p-value ranking are listed in Table 3. Interestingly, older and young patients clustered in two relatively distinct groups in a principal component analysis incorporating these top 50-ranking miRNAs (Fig. 2A), whereas patients were randomly scattered when PCA analysis was performed with the bottom 50-ranking miRNAs (Fig. 2B). The dendrogram in Fig. 3 also clearly demonstrates a high degree of clustering of the patients from both age groups. This indicates that there are indeed important age-related differences in circulating miRNA profiles. Some miRNAs showed an age-related increase in abundance, while others were found to be down-regulated in older, as compared to young, patients. When considering the 15 miRNAs exhibiting the most pronounced age-related changes (lowest p-values), increased abundance in serum of older *versus* young patients was noted for miR-21, miR-210, miR-320b, miR-378 and miR-423-5p (Fig. 4). Conversely, circulating levels of let-7d, miR-20a-3p, miR-301a, miR-374a, miR-376a, miR-191, miR-200c, miR-30b-5p, miR-140b-5p and miR-106b were decreased in older compared to young patients (Fig. 4).

**Table 3:** Exploratory serum/plasma miRNA panel screening of circulating miRNAs in older (N = 10) *versus* young (N = 10) breast cancer patients.

miRNA^a^	Age-related change^b^	Fold change	t-test P value
hsa-miR-320b	up	2.10	0.0003
hsa-miR-301a	down	3.34	0.0012
hsa-miR-210	up	2.48	0.0024
hsa-miR-21	up	2.32	0.0025
hsa-miR-376a	down	3.24	0.0036
hsa-miR-378	up	2.87	0.0037
hsa-miR-374a	down	2.13	0.0038
hsa-miR-423-5p	up	1.66	0.0044
hsa-miR-20a-3p	down	8.64	0.0049
hsa-let-7d	down	2.33	0.0052
hsa-miR-191	down	1.49	0.0061
hsa-miR-200c	down	4.88	0.0063
hsa-miR-30b-5p	down	1.78	0.0063
hsa-miR-140-5p	down	2.00	0.0064
hsa-miR-106b-5p	down	2.17	0.0097
hsa-miR-382	down	2.66	0.0109
hsa-miR-495	down	2.81	0.0132
hsa-miR-199a-3p	down	1.80	0.0141
hsa-miR-146a	up	1.83	0.0203
hsa-miR-15b*	down	2.31	0.0213
hsa-miR-320a	up	5.17	0.0218
hsa-miR-551b	down	7.10	0.0227
hsa-miR-766	up	2.24	0.0244
hsa-miR-409-3p	down	4.82	0.0261
hsa-miR-543	down	3.75	0.0308
hsa-miR-10b	up	2.06	0.0328
hsa-miR-34a	up	2.36	0.0367
hsa-miR-331-3p	down	2.37	0.0368
hsa-miR-423-5p	up	5.41	0.0383
hsa-miR-222	up	2.43	0.0410
hsa-miR-199a-5p	down	2.13	0.0436
hsa-miR-33a	up	2.09	0.0451
hsa-miR-92a	up	2.06	0.0470
hsa-miR-324-5p	down	1.51	0.0471
hsa-miR-130a	down	1.44	0.0474
hsa-miR-151-5p	down	1.39	0.0484
hsa-miR-486-5p	up	2.50	0.0489
hsa-miR-150	up	2.16	0.0511
hsa-miR-126	up	2.38	0.0568
hsa-miR-342-3p	up	1.94	0.0703
hsa-miR-15a	up	1.60	0.0759
hsa-miR-30e	up	1.29	0.0823
hsa-miR-424	up	3.15	0.0825
hsa-miR-660	up	1.53	0.0899
hsa-miR-10a	up	1.69	0.0924
hsa-miR-885-5p	down	2.44	0.0943
hsa-miR-30c	down	1.53	0.0948
hsa-miR-324-3p	up	2.07	0.0963
hsa-miR-152	up	1.58	0.1003
hsa-miR-326	down	1.83	0.1055

a This table only shows the top-50 ranking miRNAs for differential expression according to t-test. A list of all miRNAs included in the entire panel is shown in File S2.

b up : higher plasma levels found in older compared to young subjects; down : lower plasma levels found in older compared to young subjects.

### Identification of candidate reference miRNAs via miRNA serum/plasma panel screening

A second purpose of the initial screening experiment was the identification of stably expressed miRNAs that could serve as valuable references for normalisation in future validation experiments. As subsequent experiments will probably only include a limited number of selected miRNAs, global mean normalisation, requiring at least 50 genes, will not be possible anymore. To this end, the 10 miRNAs most resembling the behaviour of the global mean, i.e. the miRNAs having least variation after global mean normalisation, were selected as candidate reference miRNAs (Table 4). From these selected candidates, miR-191 was excluded since it ranged within the top 15 of age-related differential expression in the panel screening (see Table 3). The remaining 9 miRNAs, i.e. let-7i, miR-484, miR-29a, miR-29c, miR-140-3p, miR-30e, miR-30d, miR-29b and miR-590-5p, were further evaluated by the use of Normfinder [26] and GeNorm [27], two popular algorithms to select the most stably expressed reference genes from a set of tested candidate genes in a given sample panel. Normfinder, which basically calculates for each candidate reference gene the standard deviation from the global average expression (and thus mirrors the variance after global mean normalisation), yielded the following stability order: let-7i>miR-140-3p> miR-29c>miR-29a>miR-484> miR-30e>miR-29b>miR-30d>miR-590-5p. The respective stability values ranged between 0.27 for let-7i and 0.56 for miR-590-5p, with superior stability indicated by the lowest value (Table 4). Normfinder analysis also indicated that 4 is a reasonable number of normalisation genes: accumulated standard deviation values were 0.27, 0.21, 0.18, 0.16, 0.16, 0.15, 0.15, 0.14 and 014 when the number of normalizing genes was increased from just 1 to 9. Thus, the inclusion of a fourth reference gene still afforded a substantial reduction of variation retained in the dataset, while additional reference genes only contributed a minor further improvement. GeNorm analysis, which is based on a different mathematical approach, resulted in a slightly different stability ranking: miR-29a = miR-29c>miR-140-3p>let-7i>miR-29b>miR-484> miR-30e>miR-30d>miR-590-5p. The corresponding M-values (gene stability measure) increased from 0.31 for let-7i to 0.59 for miR-590-5p, again with the most stably expressed genes having the lowest M-values (Table 4). Taken together, both algorithms pointed at let-7i, miR-29a, miR-29c and miR-140-3p as the common best reference miRNAs for normalisation, which all had GeNorm and Normfinder stability values below the 0.50 cut-off for suitable reference genes [26], [27]. However, we decided not to retain let-7i since it was previously reported to be involved in genome-wide miRNA signatures of human longevity [28] and might thus be somehow age-related, which would not be desirable for the current study. Based on the above-described results, we instead selected miR-484 as the fourth reference gene.

**Table 4:** Candidate reference miRNAs identified by exploratory serum/plasma miRNA panel screening.

miRNA	Variance^a^	Normfinder	GeNorm
	after GMN	SD^b^	M-value^c^
hsa-let-7i	0.09	0.27	0.40
hsa-miR-484	0.10	0.44	0.49
hsa-miR-29a	0.18	0.36	0.31
hsa-miR-29c	0.20	0.34	0.31
hsa-miR-140-3p	0.22	0.32	0.35
hsa-miR-30e	0.23	0.46	0.53
hsa-miR-30d	0.24	0.51	0.56
hsa-miR-191	0.25	–	–
hsa-miR-29b	0.26	0.50	0.45
hsa-miR-590-5p	0.28	0.57	0.59

a Variance after global mean normalisation (GMN) : reflects the deviation of a specific miRNA from the behavior of the global mean (i.e. average signal of all miRNAs included).

b Standard deviation returned by NormFinder algorithm.

c Expression stability measure (M) returned by GeNorm algorithm (the lower, the more stable the expression).

### Validation of age-associated miRNAs

To further examine potential aging miRNAs, we focussed on the 15 miRNAs that emerged from the pilot study as those showing the most significantly different expression levels in young *versus* older individuals. For this validation study, a new independent breast cancer patient cohort was selected that basically consisted of a young group (<45 years, N = 10) and an older group (>70 years, N = 20). The latter comprised a subgroup (N = 10) of ‘fit’ older breast cancer patients and a second subgroup (N = 10) of ‘frail’ patients. These two subcategories were included to investigate whether the observed age-associated differences in plasma miRNA levels were truly related to the aging process itself (i.e. pure “aging biomarkers”), or were caused by age-related pathologies and functional decline (i.e. “frailty biomarkers”).

Individual PCR assays were carried out for each of the selected candidate aging miRNAs (i.e. miR-320b, miR-301a, miR-210, miR-21, miR-376a, miR-378, miR-374a, miR-423-5p, miR-20a-3p, let-7d, miR-191, miR-200c, miR-30b-5p, miR-140-5p and miR-106b), along with the 4 selected normalisation miRNAs (i.e. miR-29a, miR-29c, miR-140-3p and miR-484). In addition, miR-451 and miR-23a-3p were also included for hemolysis control (see Materials and Methods section). Comparable RNA extraction and reverse transcription efficiency across all 30 samples of the validation cohort was ascertained by the UniSp6 spike-in control: standard deviation of the UniSp6 PCR Cp values (after interplate calibration) was ≤0.37 cycles (mean Cp value was 20.48 cycles).

Analysis of the data first confirmed miR-29a, miR-29c, miR-140-3p and miR-484 as reliable normalisation references, all GeNorm M-values being lower than 0.5 (data not shown). However, miR-23a-3p was additionally identified as an excellent reference miRNA. This is not surprising; miR-23a-3p is incorporated in the hemolysis control test because of its stable expression in serum/plasma samples and is often used as a suitable reference miRNA for normalisation. Hence, we decided to add it to our reference miRNA panel, which thus finally consisted of 5 miRNAs.

When comparing older (N = 20) with young (N = 10) patients, significantly *decreased* miRNA plasma levels were confirmed for miR-20a-3p, miR-301a, miR-374a, miR-30b-5p, miR-106b-5p and miR-191, whereas let-7d, miR-140-5p, miR-200c and miR-376a did not reach statistical significance at the 5% significance level (Table 5). When applying Bonferroni correction for multiple testing, p-values for miR-20a-3p, miR-30b-5p, miR-106b-5p, miR-191 and miR301a were still below the corrected significance threshold of 0.00341 (Table 5).

**Table 5:** Validation of differentially expressed miRNAs in older (N = 10) *versus* young (N = 20) breast cancer patients and comparison between older ‘fit’ (N = 10) and older ‘frail’ (N = 10) subjects.

miRNA	Older vs. young		Older ‘fit’ vs. ‘frail’
	P-value^a^	Fold change^b^	P-value^a^
*Decreased expression*			
hsa-let-7d	*0.1083*	1.59	0.7861
**hsa-miR-20a-3p**	0.0004*	**2.48**	0.7405
**hsa-miR-30b-5p**	<0.0001*	**2.47**	0.8428
**hsa-miR-106b-5p**	0.0001*	**1.56**	0.8784
hsa-miR-140-5p	*0.8431*	1.06	*0.2413*
**hsa-miR-191**	0.0010*	**2.23**	0.5611
hsa-miR-200c	*0.5235*	1.21	0.5097
**hsa-miR-301a**	0.0024*	**2.34**	0.6111
**hsa-miR-374a**	**0.0109**	**2.36**	0.6765
hsa-miR-376a	*0.4679*	1.23	0.4195
*Increased expression*			
hsa-miR-21	0.4133	1.12	0.8749
hsa-miR-210	*0.1528*	1.29	*0.3447*
hsa-miR-320b	0.0585	1.25	0.6679
**hsa-miR-378**	**0.0077**	**1.71**	0.2817
hsa-miR-423-5p	0.0566	1.34	0.7050

a P-values are derived from parametric t-test (unpaired, 2-tail, C.I = 95%), unless data are not normally distributed (values indicated *in italic*). In such cases, P-value derived from Mann-Whitney test is displayed. **Bold values** indicate significant values according to the 5% significance threshold. Values indicated with an asterisk (*) remained significant after correction for multiple testing.

b Negative values indicate x-fold *decreased* expression, positive values indicate x-fold *increased* expression.

**Figure 5:**
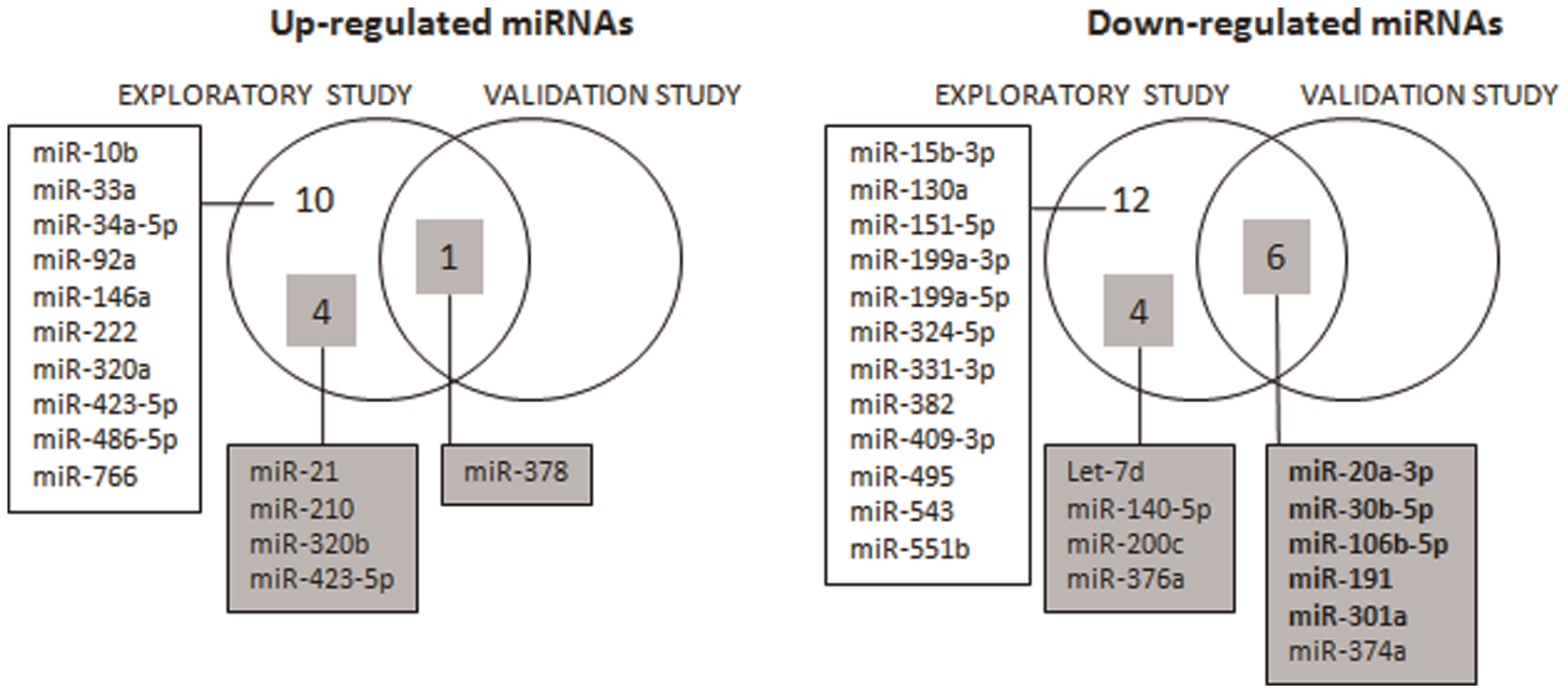
Venn diagram showing up-regulated and down-regulated miRNAs in the exploratory screening and the validation study. In the exploratory screening, there were in total 37 circulating miRNAs exhibiting age-related expression (p<0.05). Of those, only the top-15 miRNAs with the lowest p-values (shown in grey boxes) were further investigated in the validation study. Seven age-related miRNAs were confirmed in the validation study (p<0.05), of which 5 (all showing decreased expression) remained significant after correction for multiple testing; these are indicated in bold. Obviously, the right-hand zone (i.e. miRNAs showing altered expression in the validation study but not the exploratory screening) cannot contain any miRNAs, since the validation study only included those miRNAs that emerged from the exploratory study as the most significantly age-related ones.

*Increased* expression in older compared to young subjects was only confirmed for miR-378 (p = 0.0077) and was close to the 5% significance threshold for miR-320b (p = 0.0585) and miR-423-5p (p = 0.0566), while miR-21 and miR-210 were not close to statistical significance in the validation experiment. However, none of these miRNAs showing apparently *increased* expression in older subjects remained significant after Bonferroni correction for multiple testing (Table 5). An overview of upregulated and downregulated miRNAs in the exploratory screening and the validation study is shown in Fig. 5.

Interestingly, no significant differences were revealed in a subanalysis comparing the ‘fit’ and ‘non-fit’ subgroups of the older cohort for any of the microRNAs included in this validation study (all p>0.1) (Table 5). These results indicate that the observed differences in plasma miRNA levels between young and older breast cancer patients reflect a pure aging effect and do not result from age-associated changes in the patient’s global health status.

## Discussion

MicroRNA’s circulating in plasma are particularly interesting as easily accessible biomarkers for aging and age-related diseases. However, relatively little data are presently available on age-related changes in miRNA expression that can directly be monitored in human plasma. A previous study in humans has compared the miRNA expression profile in peripheral blood mononuclear cells from older versus young individuals and revealed significant age-associated down-regulation of miR-24, miR-103, miR-107, miR-128, miR-130a, miR-155, miR-221, miR-496 and miR-1538 [29], but Hunter et al. reported marked differences in miRNA expression between the peripheral blood mononuclear cells and plasma fractions of human blood [30]. In order to identify plasma microRNAs implicated in the aging process, we have compared circulating microRNA’s between young and older subjects, by applying a comprehensive RT-qPCR platform for focused microRNA profiling on serum/plasma, using samples from the large blood bank of breast cancer patients that has been established at our institution. We have chosen to perform this search for aging miRNAs in a female breast cancer population, on the plasma samples readily available from our biorepositories, in view of our eventual aim to investigate the impact of chemotherapeutic treatments on the biological age of older breast cancer patients.

For 5 miRNAs, i.e. miR-301a, miR-20a-3p, miR-106b-5p, miR-191 and miR-30b-5p, plasma expression levels were convincingly confirmed to significantly correlate with chronological age. Six other miRNAs that were selected from the intial screening (i.e. let-7d, miR-21, miR-140, miR200c, miR-210 and miR-376a) showed no significant differences between young and older patients in the validation experiment, two (i.e. miR-320b and miR-423-5p) were close to the 5% significance level and two (i.e. miR-374a and miR-378) actually reached statistical significance, but were not retained after correction for multiple testing. Nevertheless, each of the miRNAs included in the validation study consistently showed the same trend, regarding decreased or increased expression in older *versus* young subjects, as observed in the exploratory screening. It should be kept in mind that patient numbers were rather small in both the initial screening and the validation. This might explain why several miRNAs, emerging as candidate aging miRNAs from the exploratory experiment, did not reach statistical significance in the subsequent validation step. On the other hand, we neither can exclude the possibility that for these miRNAs, the ‘differences’ initially observed in the screening study did not reflect ‘real’ differential expression but were merely coincidental findings due to multiple testing. Another aspect that may explain inconsistencies between the screening and validation steps is the use of different specimens : because of sample availability issues, the validation study was done on plasma, while serum was used in the initial screening. Nevertheless, one can argue that if the same five miRNA were found to be differentially expressed between age categories in both serum and plasma, regardless of the preparation procedure, these can be considered as particularly strong biomarkers.

It is well possible that we ‘missed’ significant aging-related miRNAs, either because they are not included in the initial screening panel (which contains a selection of the 175 most commonly detected circulating miRNAs out of the ∼300–400 miRNAs that have ever been annotated to serum/plasma), or because they were not among the top-15 ranking differentially expressed miRNAs in the pilot study and were thus not selected for further investigation, future studies will not exclusively be limited to the five aging miRNAs identified here. For instance, we have observed significantly increased expression of miR-34a in older compared to young subjects in our exploratory screening, which is in line with previous findings [22]. However, miR-34a was not included in our validation experiment because for the present study, we have chosen for an unbiased, straight-forward selection of the top 15 differentially expressed miRNAs to be included in the validation study. We are currently starting a miRNA profiling study including miR-34a in older breast cancer patients receiving chemotherapy.

Interestingly, of the 15 potential aging-related miRNAs selected from the initial screening, 8 miRNAs (i.e. let-7d, miR-106b-5p, miR-20a-3p, miR-21, miR-301a, miR-320b, miR-374a and miR-423-5p) were previously reported to be associated with healthy longevity in a recent study by El Sharawy et al. on genome-wide miRNA signatures [28]. In line with our current findings, this study also revealed age-related down-regulation of let-7d, miR-106b-5p, miR-20a-3p, miR-301a and miR-374a, while miR-320b and miR-423-5p were shown to be upregulated with increasing age. The 7 other potential aging miRNAs that were selected from our initial screening, i.e. miR-140-5p, miR-200c, miR-210, miR-376a, miR-378, miR-191, miR-30b-5p, were not reported to show age-related changes in the longevity study, although the latter two miRNAs were definitely confirmed in our validation study. On the other hand, several miRNAs showing age-related differential expression in the longevity study were also included in our initial screening panel but did not show significant differences between young and older patients and were therefore not further investigated. However, the different context of both studies must be underscored : while the study by El Sharawy and coworkers refers to healthy aging and longevity, the age-related miRNAs reported here are found in a breast cancer background. Moreover, both studies were conducted on relatively small patient cohorts, each containing only ≤20 older patients. Thus, it is well possible that they both lacked sufficient statistical power to detect subtle differences between young and older patients. This, together with the use of different experimental methodology and statistical thresholds, may account for the partial discrepancies. Nonetheless, it is quite remarkable that such highly significant age-related changes in circulating miRNAs, as observed in our study for miR-301a, miR-20a-3p, miR-106b-5p, miR-191 and miR-30b-5p, can be evidenced with as little as 10 patients per age group, suggesting that these miRNAs can be considered as robust and reliable aging biomarkers.

Several of the top-ranking miRNAs for age-related differences between young and older breast cancer patients in our exploratory study have already been linked with cellular senescence in previous reports. A study using diverse human cellular as well as organismal aging models revealed miR-17, miR-19b, miR-20a and miR-106a as biomarkers of aging [31]. These miRNAs, which all belong to the oncogenic miR-17∼92 cluster [32], [33], prevent cellular senescence by down-regulating the cell cycle inhibitor p21/CDKN1A. Likewise, family members of the paralog miR-106b∼25 cluster also promote cell cycle progression − and thus inhibit senescence − by directly targeting p21/CDKN1A [34]. Consistent with these findings, we observed a highly significant age-related decrease in plasma levels of miR-20a and miR-106b, which might thus correspond to an increased level of senescence. Notably, these two miRNAs have previously also been reported to be down-regulated in peripheral blood mononuclear cells from octogenarians *versus* young people, whereas centenarians exhibiting a healthy aging phenotype seemed capable of maintaining high levels of these senescence inhibitors [35]. We found no evidence, however, pointing to miR-17, miR-19b or miR-106a as circulating aging markers, although these miRNAs were also included in the serum/plasma screening panel used in our exploratory study. Neither did we find age-related changes in plasma expression of miR-24, which has been shown to suppress the expression of the senescence gene p16^INK4A^ in human diploid fibroblasts [36]. As opposed to the senescence inhibiting miRNAs miR20a and miR-106b, 6 other miRNAs that were identified as candidate circulating aging miRNAs in our initial exploratory study (i.e. miR-21, miR-191, miR-200c, miR-210, miR-376a and let-7d) have been reported to *trigger* cellular senescence in specific cell types [20], [37]–[40]; miR-21 has even been described as a new circulating marker of inflammaging [41]. Yet, only miR-191 was convincingly confirmed in our validation study; its significant age-associated down-regulation in serum/plasma is in agreement with earlier findings in mononuclear cells [35]. The remaining 7 candidate aging miRNAs miR-320b, miR-301a, miR-378, miR-374a, miR-423, miR-30b, miR-140 that emerged from our initial screening and of which two (i.e. miR-301a and miR-30b) were convincingly confirmed in the validation study, have previously not been linked with cellular senescence and are now for the first time directly associated with aging in humans. It should be mentioned, though, that one of the target genes of miR-320 family members is IGFBP5 (insulin-like growth factor binding protein 5), an important modulator of the aging-related insulin/IGF-1 signaling pathway. IGFBP5 and miR-320 are expressed in a wide variety of overlapping tissues (e.g. liver, brain, lung, kidney, heart, colon …) and are secreted into the circulation. Serum IGFBP5 concentrations have been reported to decline with age and to show a significant positive correlation with circulating levels of IGF-1 [42]. Thus, the age-associated increase in circulating miR-320b observed in our present study – albeit not statistically significant in the validation experiment – might possibly be linked with our previous finding that plasma IGF-1 levels are decreased in older compared to younger patients (our own data, manuscript in preparation). Therefore, miR-320b will still be considered as a potential miRNA of interest in our future aging biomarker studies.

For reasons outlined above, we are not studying aging miRNAs in a healthy population but in a breast cancer context. In order to minimize cancer-related confounding effects, we have selected highly homogeneous cohorts with regard to tumor characteristics. Nevertheless, it cannot be excluded that the organismal response to breast cancer disease might override otherwise regulated miRNAs. Small age-related changes in plasma miRNA expression might possibly be masked by substantial cancer-related under- or overexpression of particular miRNAs in older breast cancer patients. The processes of aging and carcinogenesis are indeed intimately linked, since cellular senescence constitutes an intrinsic tumor suppressor mechanism *in vivo* but, at the same time, is the major driving force in age-related tissue degeneration and –dysfunction [43]. It is thus conceivable that miRNAs implicated in aging are also modulated in human cancers and *vice versa*. Several miRNAs that emerged from our exploratory screening as candidate aging miRNAs have actually been reported as “oncomirs” or “tumor suppressor miRNAs”. For instance, let-7d is considered a tumor suppressor microRNA, as it is strongly down-regulated in poorly differentiated lung and breast tumor cells [44]. Also, miR-376a is downregulated in several tumors and makes part of a miRNA cluster frequently silenced in malignant melanoma [45]. In contrast, miR-20a, miR-21, miR-378, miR-106b and miR-191 were found to exert oncogenic activity and are overexpressed in several types of cancer [20], [31], [34], [46], [47]. Moreover, high (tumor) expression of miR-210 has been associated with higher risk of recurrence in breast cancer [48]. Serum miR-200c was reported as a novel prognostic and metastasis-predictive biomarker in colorectal cancer [49], while miR-423-5p was found to be upregulated in serum from patients with gastric cancer [50]. Therefore, it should be kept in mind when interpreting our data that certain miRNAs exert their action at the interface between cancer and aging. To our knowledge, this is the first miRNA study comparing “fit” and “non-fit/frail” older individuals. We did not observe any significant differences between those two subgroups, at least not with regard to the miRNAs investigated in the validation study. This is somewhat surprising, since several of these miRNAs have also been implicated in age-related diseases. For instance, let-7d, miR-191 and miR-301a make part of a unique circulating 7-miRNA signature that can distinguish patients with Alzheimer’s disease from normal controls [51], and miR-20a and miR-106b are both implicated in transcriptional inhibition of Alzheimer’s amyloid precursor protein (APP) [52]. Also, miR-320 was shown to be upregulated in neurodegeneration in mice [53]. Furthermore, miR-21 expression has been reported to be higher in patients suffering from cardiovascular disease [41] and loss of miR-140 contributes to the development of age-related osteoarthritis-like changes in mice [54]. However, other miRNAs than those included in our validation study have been more consistently associated with age-related physical and mental decline: e.g. the miRNA most clearly associated with age-related neurodegeneration in the aging *Drosophila* brain is miR-34 [55]. Our data indicate that the observed age-related differential expression of the 5 validated aging miRNAs represents a direct effect of physiological aging and is not merely associated to the age-related frailty syndrome. Yet, the newly identified aging miRNAs should be further validated in larger cohorts of frail *verus* non-frail older persons that are not affected by cancer, in order to ultimately sustain this conclusion. A search for “frailty miRNAs” is another issue of clinical interest, which would, however, demand a different experimental set-up, such as exploratory screening and subsequent validation in healthy *versus* frail subjects of comparable age.

In conclusion, we have discovered five miRNAs (miR-301a, miR-20a-3p, miR-106b-5p, miR-191 and miR-30b-5p) that can be used as aging biomarkers in future research projects. Since we have chosen for a straight-forward selection of 15 miRNAs from the initial screening, certain potentially interesting miRNAs, most particularly miR-34a, were not further investigated in this study. These, together with several miRNAs that remained inconclusive because of borderline statistical significance (e.g. miR-320b, miR-374a, miR-378, miR-423), might yet merit further attention in future studies. The newly identified aging miRNAs will now be examined in a prospective study that has recently been conducted in our institution with the aim to investigate the impact of chemotherapy on the biological age of older breast cancer patients. While common acute adverse events of chemotherapy, such as neutropenia and mucosal degeneration, are well-known and may be tolerated/accepted by the patient, the long term impact of cytotoxic treatments (and perhaps other anti-cancer therapies such as hormonal drugs, monoclonal antibiodies and oral targeted agents) on healthy tissues is actually poorly documented. For instance, premature telomere shortening due to DNA-targeting chemotherapeutic drugs may impact on the long-term replicative potential of regenerative tissues like hair follicles, the hematopoietic and gastrointestinal system, and germline and skin cells [56]. To learn more about such delayed aging-related side effects in long-term survivors of cancer, a better insight into aging mechanisms and associated biomarkers is mandatory, along with detailed investigation of the impact of common chemotherapeutic regimens on these aging biomarkers.

## Materials and Methods

### Ethics statement

This study was performed in compliance with the Helsinki Declaration and national law. All patients included in the study gave written informed consent for future translational research. Blood sampling, collection of patient data and genetic analysis were approved by the ethics committee of our institution (Ethics Committee of the University Hospitals Leuven, study number S53608; Approval number : ML7994).

### Patients

Since 2003, the Leuven Multidisciplinary Breast Center (University Hospitals Leuven) has systematically collected plasma and serum from all consenting (i.e. ±75%) breast cancer patients at the time of diagnosis, before initiation of any local or systemic therapy. All breast cancer patients are also included in a clinical database, containing extensive general and tumor-related information, as well as clinical follow-up. In addition, most older (70+) breast cancer patients diagnosed since 2009 are also included in one of the large-scale geriatric cancer projects running in our institution: geriatric assessment (GA) − integrating two geriatric screening tools G8 and Flemish version of the Triage Risk Screening Tool (fTRST), Activities of Daily Living (ADL), instrumental Activities of Daily Living (iADL), Geriatric Depression Scale, Mini Mental State Examination, and Mini Nutritional Assessment − is systematically performed prior to initiation of any chemotherapy, radiotherapy or surgery [24]. Furthermore, the Charlson Comorbidity Index (at the time of diagnosis) can easily be calculated retrospectively using the patient’s electronic file.

### Selection of the exploratory cohort

For an initial exploratory pilot experiment, eligible subjects were selected from the clinical breast cancer database based on the following inclusion criteria: (i) diagnosed with primary (i.e. non-relapse) early (i.e. non-locally advanced, non-metastatic) invasive breast cancer *without* lymph node involvement; (ii) received primary surgery and pathological confirmation in our institution; (iii) serum collected at the time of diagnosis (i.e. before initiation of any treatment); (iv) all pathological parameters available to identify histological subtype according to recent guidelines [57], [58]. A study group of 10 young (<45 years) patients and 10 older (>70 years) patients (all female), the latter with GA performed at the time of diagnosis (i.e. before initiation of any treatment), was selected and both age groups were matched in order to contain an equal distribution of different breast cancer subtypes: 7 so-called luminal A, 1 luminal B, 2 triple negative [58]. Tumor characteristics are summarized in Table 1. Determination of tumor grading and estrogen receptor (ER), progesterone receptor (PR), and HER2/*neu* status was done according to standard procedures. ER and PR were considered positive if >1% of cells stained positive on immunohistochemistry. HER2 was considered positive if the fluorescent in situ hybridization (FISH) test, systematically performed in all immunohistochemistry (IHC) 2+ tumors, showed HER2 genomic amplification or, in the absence of FISH, if IHC was 3+.

### Selection of the validation study cohort

For the subsequent validation experiment, an independent cohort of early breast cancer patients (all with primary invasive, node-negative luminal A tumors) was selected consisting of 10 young (<45 years) female patients and 20 older (>70 years) female patients, from whom 10 were ‘fit’ while 10 others were classified as ‘non-fit’. Fitness/frailty status was estimated based on the results of the GA, which were condensed into (i) the generally used Balducci score [59] and (ii) the novel Leuven Oncogeriatric Frailty Score (LOFS), a final score from 0 (severly frail) to 10 (entirely fit), described in File S1. Patients of the older ‘fit’ group had LOFS = 9–10, whereas patients of the older ‘non-fit’ group had LOFS ≤5. Clinical characteristics of patients from the validation study cohort are summarized in Table 2.

### Serum and plasma collection

Peripheral blood was routinely sampled in 4-mL BD Vacutainer SST II Advance tubes (for serum collection) and in 4-mL BD Vacutainer EDTA K2E tubes (for plasma collection). The blood samples were incubated at room temperature for 20 to 60 min. After centrifugation at 1600xg for 10 min at 4°C, the supernatant (serum/plasma) was isolated and stored in aliquots at −80°C. Because of sample availability issues, the validation study was done on plasma specimens, whereas the exploratory screening was done on serum.

### MicroRNA isolation

After spinning down thawed serum/plasma to remove debris, the absorbance spectrum of the sample was recorded on a NanoDrop ND-1000 in order to detect hemolysis, which may cause severe perturbation of serum/plasma miRNA profiles due to cell-derived microRNA contamination. Samples showing a clear absorption peak at 415 nm (i.e. absorbance maximum of hemoglobin) were excluded from the experiment. Absence of hemolysis in the included samples was further verified after qPCR (see below). RNA was purified from non-hemolysed serum/plasma samples using the miRNeasy Mini kit (Qiagen) following the manufacturer’s protocol with slight modifications in order to achieve optimal microRNA extraction from serum/plasma samples, which typically contain very low amounts of RNA. Briefly, to 200 µL of serum/plasma, 1 µg of carrier MS2 RNA (Roche) was added, together with 750 µL of QIAzol (Qiagen). Subsequently, 1 µL of a synthetic RNA spike-in (UniSp6 at 10^8^ copies/µL, Exiqon) was added to allow evaluation of the efficiency and uniformity of the entire RNA extraction/cDNA synthesis procedure. After 5 min of incubation, 200 µL chloroform was added. After incubation and centrifugation according to the normal procedure, the upper aqueous phase was isolated, 1.5 volumes of ethanol was added and the sample was loaded onto the spin column. The column was washed once with RWT buffer and three times with RPE buffer (both included in the kit). After air-drying of the column, the RNA sample was eluted with 50 µL of nuclease-free water and immediately frozen at −80°C until further analysis. In both the pilot and the validation experiment, duplicate RNA extractions were performed in parallel from each plasma sample.

### cDNA synthesis

Because of the very low amounts of RNA in serum/plasma and, therefore, the use of an RNA carrier during the extraction procedure, standard spectrophotometric measurement of RNA yield and quality is not suitable for serum/plasma RNA extracts. Therefore, RNA input in the reverse transcription (RT) reaction was based on sample volume and not RNA quantity. Preliminary test experiments with several commonly used control miRNAs (i.e. miR-103, miR-191, miR-423-3p and miR-451) were carried out to determine the optimal amount of RNA template to be used in the RT reaction; these titration experiments showed that an RNA input of 4 µL mostly resulted in good RT efficiency and subsequent PCR amplification without significant interference by RT and/or PCR inhibitors, which are frequently present in RNA preparations from serum/plasma. Thus, cDNA was synthesized from each of both duplicate RNA extracts using 4 µL of RNA in a 20-µL reaction by use of the Universal cDNA synthesis kit II (Exiqon) according to the manufacturer’s instructions. cDNA samples were stored frozen until PCR analysis was performed.

### RT-qPCR-based miRNA assays

In the initial experiment, a broad exploratory miRNA screening was performed using the 96-well Serum/Plasma Focus microRNA PCR Panel (Exiqon) that comprises microRNA PCR assays for 175 different microRNAs that are commonly found in serum/plasma. Since individual samples are assessed on separate plates, the panel assay also includes triplicate wells of an interplate calibrator (UniSp3) to account for technical run-to-run differences in amplification signal. Data from different plates were normalized using the Cp-values of the interplate calibrator. For each serum sample, both duplicate cDNA samples were pooled before running the miRNA panel assay. Assays were carried out following the manufacturer’s protocol. Briefly, pooled cDNA was diluted 50x in nuclease-free water and mixed with an equal volume of 2x SYBR Green master mix (Exiqon). Final reaction volume was 10 µL. Plates were run on a LightCycler 480 (LC480, Roche) instrument applying the following thermal cycling protocol : activation step (10 min at 95°C); 45 amplification cycles (10 s at 95°C, 1 min at 60°C, ramp rate 1,6°C/s); melting curve analysis.

In the subsequent validation study on plasma samples from a different, independent patient cohort, 21 selected miRNAs were assessed by the use of individual PCR assays (MicroRNA LNA PCR primer sets, Exiqon). These included the 15 top-ranking candidate aging miRNAs, showing the most pronounced age-related differences in the pilot screening experiment. In addition, 4 miRNAs that were identified as good reference candidates in the pilot experiment and 2 additonal quality control miRNAs were also included. For each plasma sample, duplicate RNA extracts were prepared and reverse transcribed and separate PCR assays were run on each of both duplicate cDNA samples. All PCR assays were done in triplicate microplate wells (96-well format). Reaction mixtures were prepared according to the provided assay protocol and contained 4 µL of 40-fold diluted cDNA (20-fold diluted for miR-20a-3p) in a final volume of 10 µL. For each miRNA, all samples were run together on the same plate to avoid bias introduced by plate-to-plate variations in qPCR efficiency. Plates were run on the LC480 instrument using the same thermal cycling protocol as described above for the panel assays.

### Data analysis

RT-qPCR Cp values were determined by the LC480 instrument software using the second derivative method and were subsequently imported and further processed by GenEx Pro software (MultiID Analyses). The absence of hemolysis in the initial serum/plasma samples was verified by means of the miR-451/miR-23a-3p hemolysis test: ΔCp values between miR-23a-3p, known to be stably expressed in serum/plasma samples, and miR-451a, known to show highly increased expression in hemolysed samples, were below 5 for all samples.

Data of the initial screening experiment were normalized using the global mean of the entire miRNA panel [25]. Candidate reference miRNAs were then identified by variance analysis of the normalized dataset and were further examined using the specified algorithms GeNorm [27] and Normfinder [26], which are both incorporated in the GenEx Pro software package (MultiID Analyses). Five miRNAs with superior expression stability among all samples were used for data normalisation in the subsequent validation study.

Statistical tools incorporated in the GenEx software package were applied to assess differences in serum/plasma miRNA expression between the different patient groups. These included parametric t-test (unpaired, two-tailed) for normally distributed miRNA results and Mann-Whitney test (unpaired, two-tailed) for cases where the data were not normally distributed. Normality was assessed with the Kolmogorov-Smirnov test. In the exploratory study, principal component analysis and hierarchical clustering were additionally applied to reveal sample grouping patterns in the multivariate dataset. In the validation study, the age effect was first assessed by comparing the young patient group (N = 10) with the total older patient group (N = 20), combining both ‘fit’ (N = 10) and ‘frail’ (N = 10) subgroups. Subsequently, a comparison between ‘fit’ and ‘frail’ patients was done within the older cohort. Correction for multiple testing was done according to the Dunn-Bonferroni method.

## Supporting Information

File S1 **Leuven Oncogeriatric Frailty Score (LOFS).** Description and illustration of the composition of the new, refined scoring system that was used to evaluate the fitness/frailty status of the patients.
(DOCX)

File S2 **List of all microRNAs included in the exploratory screening panel.**
(DOCX)

File S3 **Data set of the exploratory screening study.**
(XLSX)

File S4 **Data set of the validation study.**
(XLSX)
