# Supplementary material for: Circulating MicroRNAs as Easy-to-Measure Aging Biomarkers in Older Breast Cancer Patients: Correlation with Chronological Age but Not with Fitness/Frailty Status
Source: PLoS One. 2014 Oct 21;9(10):e110644. doi: 10.1371/journal.pone.0110644 (PMC4204997; doi:10.1371/journal.pone.0110644)
Supplement: File S1 — Leuven Oncogeriatric Frailty Score (LOFS). Description and illustration of the composition of the new, refined scoring system that was used to evaluate the fitness/frailty status of the patients. (DOCX) [file pone.0110644.s001.docx]

**S1 - Leuven Oncogeriatric Frailty Score (LOFS)**

Contribution of the individual GA test items to the final LOFS score :

ADL (6):

Maximum (6/6) independent : +2

5/6 and 4/6 independent = +1

3/6 or less independent : +0

ADL(24):

6/24 -8/24 : +2

9/24 - 12/24 : +1

13/24 - 24/24 : +0

iADL (8): vrouwen

8/8 : +2

7/8 - 4/8 : +1

3/8 – 0/8 : +0

MMSE :

30-28 : +2

27-24 : +1

23 or less : 0

MNA :

14-12 : +2

8 – 11 : +1

7 or less : 0

CCI :

No comorbidities : +2

One point at CCI : +1

Two or more points at CCI : 0

Category 0 till 10 reflects the level of frailty :

**0**

**10**

VULNERABLE

FRAIL

FIT

**Grouping of patients in categories using cut-off points is done according to the following rule :**

**10 - 9 : category 1 : Fit**

**8 – 7 : category 2 : Slightly vulnerable**

**6 – 5 : category 3 : Vulnerable**

**4 – 3 : category 4 : Frail**

**2 -0 : category 5 : Severely Frail**
