# Supplementary material for: Circulating MicroRNAs as Easy-to-Measure Aging Biomarkers in Older Breast Cancer Patients: Correlation with Chronological Age but Not with Fitness/Frailty Status
Source: PLoS One. 2014 Oct 21;9(10):e110644. doi: 10.1371/journal.pone.0110644 (PMC4204997; doi:10.1371/journal.pone.0110644)
Supplement: File S2 — List of all microRNAs included in the exploratory screening panel. (DOCX) [file pone.0110644.s002.docx]

**S2 - List of all microRNAs included in the exploratory screening panel**

(Exiqon serum/plasma focus panel)

hsa-let-7a

hsa-let-7b

hsa-let-7b*

hsa-let-7c

hsa-let-7d

hsa-let-7d*

hsa-let-7e

hsa-let-7f

hsa-let-7g

hsa-let-7i

hsa-let-7i*

hsa-miR-1

hsa-miR-101

hsa-miR-103

hsa-miR-103-2*

hsa-miR-106a

hsa-miR-106b*

hsa-miR-106b-5p

hsa-miR-107

hsa-miR-10a

hsa-miR-10b

hsa-miR-122

hsa-miR-125a-5p

hsa-miR-125b

hsa-miR-126

hsa-miR-127-3p

hsa-miR-128

hsa-miR-130a

hsa-miR-132

hsa-miR-133a

hsa-miR-133b

hsa-miR-136

hsa-miR-139-5p

hsa-miR-140-3p

hsa-miR-140-5p

hsa-miR-142-3p

hsa-miR-142-5p

hsa-miR-143

hsa-miR-144

hsa-miR-144*

hsa-miR-145

hsa-miR-146a

hsa-miR-148a

hsa-miR-148b

hsa-miR-150

hsa-miR-151-3p

hsa-miR-151-5p

hsa-miR-152

hsa-miR-154

hsa-miR-155

hsa-miR-15a

hsa-miR-15b

hsa-miR-15b*

hsa-miR-16

hsa-miR-16-2*

hsa-miR-17

hsa-miR-181a

hsa-miR-182

hsa-miR-185

hsa-miR-186

hsa-miR-18a

hsa-miR-18a*

hsa-miR-18b

hsa-miR-191

hsa-miR-192

hsa-miR-193b

hsa-miR-194

hsa-miR-195

hsa-miR-197

hsa-miR-1974

hsa-miR-199a-3p

hsa-miR-199a-5p

hsa-miR-19a

hsa-miR-19b

hsa-miR-200c

hsa-miR-205

hsa-miR-20a

hsa-miR-20a-3p

hsa-miR-20b

hsa-miR-21

hsa-miR-210

hsa-miR-2110

hsa-miR-215

hsa-miR-22

hsa-miR-22*

hsa-miR-221

hsa-miR-222

hsa-miR-223

hsa-miR-223*

hsa-miR-23a-3p

hsa-miR-23b

hsa-miR-24

hsa-miR-25

hsa-miR-26a

hsa-miR-26b

hsa-miR-27a

hsa-miR-27b

hsa-miR-28-3p

hsa-miR-28-5p

hsa-miR-297

hsa-miR-29a

hsa-miR-29a*

hsa-miR-29b

hsa-miR-29b-2*

hsa-miR-29c

hsa-miR-301a

hsa-miR-30a

hsa-miR-30b-5p

hsa-miR-30c

hsa-miR-30d

hsa-miR-30e

hsa-miR-30e*

hsa-miR-32

hsa-miR-320a

hsa-miR-320b

hsa-miR-324-3p

hsa-miR-324-5p

hsa-miR-326

hsa-miR-328

hsa-miR-331-3p

hsa-miR-335

hsa-miR-338-3p

hsa-miR-339-3p

hsa-miR-339-5p

hsa-miR-33a

hsa-miR-342-3p

hsa-miR-346

hsa-miR-34a

hsa-miR-361-3p

hsa-miR-363

hsa-miR-365

hsa-miR-374a

hsa-miR-374b

hsa-miR-375

hsa-miR-376a

hsa-miR-378

hsa-miR-382

hsa-miR-409-3p

hsa-miR-421

hsa-miR-423-3p

hsa-miR-423-5p

hsa-miR-424

hsa-miR-425

hsa-miR-425*

hsa-miR-451

hsa-miR-484

hsa-miR-485-3p

hsa-miR-486-5p

hsa-miR-495

hsa-miR-497

hsa-miR-500a

hsa-miR-501-3p

hsa-miR-502-3p

hsa-miR-505

hsa-miR-532-3p

hsa-miR-532-5p

hsa-miR-543

hsa-miR-551b

hsa-miR-574-3p

hsa-miR-584

hsa-miR-590-5p

hsa-miR-605

hsa-miR-629

hsa-miR-652

hsa-miR-660

hsa-miR-720

hsa-miR-766

hsa-miR-885-5p

hsa-miR-92a

hsa-miR-92b

hsa-miR-93

hsa-miR-93*

hsa-miR-95

hsa-miR-99a

hsa-miR-99b
